# Exploring Diagnostic Challenges: A Case Report Commentary on “Solving a Diagnostic Dilemma in a Patient With Periodic Fever—When the Pieces of the Puzzle Finally Fit”

**DOI:** 10.1002/ccr3.72289

**Published:** 2026-03-13

**Authors:** Fatih Kelesoglu, Adem Polat

**Affiliations:** ^1^ University of Pittsburgh Pittsburgh Pennsylvania USA; ^2^ Norfolk and Norwich University, West Suffolk Hospital Bury St Edmunds UK

**Keywords:** Familial Mediterranean Fever (FMF), interferonopathy, MEFV mutation, NLRP12 variant, periodic fever syndromes, type I interferon signature

## Abstract

This case highlights the diagnostic challenge of distinguishing periodic fever syndromes with prominent neurological features from interferonopathies. While the absence of specific features on MRI/MRA and SAVI reduces the likelihood of certain subtypes, genetic evaluation involving interferon pathway genes remains important. Early diagnosis can prevent complications and guide personalized treatment.

Dear Editor,

I am writing this letter to contribute to your case report titled “Solving a diagnostic dilemma in a patient with periodic fever—When the pieces of the puzzle finally fit” [1]. This case is an essential example of a clinically complex disease's diagnostic challenges and treatment options. I commend the effort put into solving this case under challenging circumstances; it reminded me of an episode from the TV series “House.” It even raises the question of why a film shouldn't be made about it.

In your case, the combination of Familial Mediterranean Fever (FMF) and neurologic symptoms presents an unusual clinical picture. In particular, neurologic symptoms are at the forefront in this patient, unlike typical FMF cases, creating confusion in the diagnostic process. Such cases with predominant neurological symptoms usually require early diagnosis and appropriate treatment; otherwise, the disease may progress and lead to severe complications.

The pathogenic variant detected in the MEFV gene (c.2230G>T, p.Ala744Ser) and the variant of uncertain significance in the NLRP12 gene (c.411C>A, p.Phe137Leu) emphasize the importance of genetic testing and raise new questions about how these genetic variants may affect the clinical course of the disease. In particular, the possibility of interferonopathy is a critical issue to consider in such patients. In particular, conditions such as Aicardi‐Goutières Syndrome (AGS) (known to be associated with systemic lupus erythematosus (SLE)), STING‐associated Vasculopathy of Infancy onset (SAVI) and Singleton‐Merten Syndrome should be evaluated. Type I interferon response should be assessed by further genetic analysis.

There are several justifications for the idea of interferonopathy:

*Neurologic symptoms*: The predominance of neurological symptoms (chronic headaches, seizures, loss of consciousness, etc.) suggests central nervous system involvement, which is common in interferonopathies.

*Chronic inflammation*: The patient's recurrent fever, skin rashes, and joint pains indicate the presence of a chronic inflammatory process. Overproduction of type I interferon in interferonopathies may trigger such chronic inflammatory responses.

*Autoimmune findings*: Laboratory findings reflecting autoimmunity, such as ANA positivity (A positive rate of 1/100 is significant) and poor ENA results, are consistent with autoimmune reactions in interferonopathies.

*Genetic findings*: The potential link of the variant in the NLRP12 gene with autoinflammatory syndromes and the contribution of the mutation in the MEFV genes to inflammatory processes are in line with the genetic irregularities observed in interferonopathies.

For these reasons, the possibility of interferonopathy should be seriously considered in this case. I also recommend Whole Exome Sequencing (WES) to clarify the diagnosis and to evaluate other potential genetic factors (e.g., TREX1, RNASEH2A, RNASEH2B, RNASEH2C, ADAR1, IFIH1, TMEM173, NLRP3, NLRP12).

We acknowledge that the absence of neuroradiological abnormalities on MRI/MRA and the lack of SAVI‐specific features (e.g., ILD or cold‐induced acral ulcers) make these diagnoses less likely. Nevertheless, interferonopathies should remain in the differential diagnosis, and comprehensive genetic testing can help exclude or confirm such possibilities.

The positive ANA test years before the onset of lupus and the activation of the interferon pathway in lupus raise the possibility of lupus in this patient. Therefore, the patient should be monitored for the potential development of lupus in the future [2, 3, 4]. I congratulate you again for your outstanding efforts and wish you success.

Best regards.

## Author Contributions

**Fatih Kelesoglu:** conceptualization, writing – original draft, writing – review and editing. **Adem Polat:** conceptualization, writing – original draft, writing – review and editing.

## Funding

The authors have nothing to report.

## Conflicts of Interest

The authors declare no conflicts of interest.

## Data Availability

This letter is based on previously published data and does not contain any new or original datasets.
